# The Paradox of the Moderate Muslim Discourse: Subtyping Promotes Support for Anti-muslim Policies

**DOI:** 10.3389/fpsyg.2020.612780

**Published:** 2020-12-21

**Authors:** Nader H. Hakim, Xian Zhao, Natasha Bharj

**Affiliations:** ^1^Department of Psychology, Furman University, Greenville, SC, United States; ^2^Rotman School of Management, University of Toronto, Toronto, ON, Canada; ^3^Ithaca College, Ithaca, NY, United States

**Keywords:** subtyping, Islamophobia, Muslims/Islam, prejudice/stereotyping, secular critique

## Abstract

Tolerant discourse in the United States has responded to heightened stereotyping of Muslims as violent by countering that “not all Muslims are terrorists.” This subtyping of Muslims—as some radical terrorists among mostly peaceful “moderates”—is meant to protect a *positive* image of the group but leaves the original negative stereotype unchanged. We predicted that such discourse may paradoxically increase people’s support of anti-Muslim policies because the subtyping and its associated negative stereotypes justify hostile actions toward Muslims. In Study 1, subtyping predicted support for three anti-Muslim policies, but only among political moderates and conservatives. In Study 2, participants who were exposed to subtyping narratives expressed greater support for surveillance of Muslims in the United States. The effect of subtyping narrative exposure was stronger on support for hawkish anti-terror policy when participants’ preexisting endorsement of subtyping was low. Irrespective of the well-meaning intentions of peaceful vs. radical subtyping, its expression can justify ongoing “War on Terror” policies. As the population of Muslims increases in North America, the intuition that most Muslims do not meet the negative stereotype may ironically reduce inclusion.

## Introduction

During the third debate leading up to the 2016 U.S. presidential election, a Muslim American asked the candidates how they would respond to rising Islamophobia. The candidates began their responses by opposing prejudice and then converged on a longstanding narrative that understands Muslims as a group with distinct responsibility for thwarting violence (Politico Staff, 2016):

Donald Trump: Well, you’re right about Islamophobia, and that’s a shame … but whether we like it or not, there is a problem. And we have to be sure that Muslims come in and report when they see something going on. When they see hatred going on, they have to report it.

Hilary Clinton: … unfortunately there has been a lot of very divisive, dark things said about Muslims … We need American Muslims to be part of our eyes and ears on our front lawns … Part of our homeland security.

Both responses opposed anti-Muslim prejudice and in so doing also reinforced a central message to the Muslim questioner and to the broader audience: a minority of Muslims in America can potentially harm, and a peaceful majority has a responsibility to stop it. Though some parts of their answers not included above certainly diverged in tone and content, it is noteworthy that the candidates responded similarly amidst such a divisive election season.

We argue that the dialogue captured in that debate is a poignant example of an intercultural stereotype of Muslims as either radical or moderate, a form of subtyping, as it called in the stereotyping literature. We argue in this paper that the subtyping of Muslims emerges within a militaristic historical period, one that produced a sociocultural framing of Muslims as peaceful but always potentially violent. Furthermore, the stereotype continues to function as a basis for building popular support for aggressive policies through its anti-prejudicial veneer.

### Subtyping Informs and Protects Stereotypes

Subtyping refers to the process of distinguishing members of a category while retaining a general stereotype about the category. Research conducted within the cognitively oriented period of the stereotyping literature proposed at least two purposes for subtyping. First, subtyping can specify examples that constitute a category that is too broad to be understood with a single stereotype (Devine and Baker, 1991). For instance, while a general schema can structure perception of African Americans in the United States, particular stereotypes animate this structure through subtypes such as “streetwise,” “athlete,” or “poor,” often to prejudicial effect (Devine and Baker, 1991). The category of *elderly* may be subtyped either as “grandmotherly” or “elder statesman,” among others (Brewer et al., 1981). Here, the subtypes inform a hierarchical perception of a unitary category and allows more diverse stereotypical representations of the group. Some scholars also label this type of subtyping as subgrouping (e.g., Richards and Hewstone, 2001), in which subgroups exist within the superordinate group and stereotypes of the superordinate group are still valid (Brown et al., 2018).

Second, and more related to the current research, subtyping can insulate a category’s general stereotype in the face of disconfirming evidence or non-stereotypical examples (Weber and Crocker, 1983; Kunda and Oleson, 1995; Queller and Smith, 2002; Joyce et al., 2020). That is, subtyping allows for individuals to be understood as unrepresentative of the broader category (Weber and Crocker, 1983). This is especially the case when deviance cannot be attributed to any other information about the target. For instance, the stereotype of gay men as promiscuous did not change in the face of disconfirming examples when the disconfirming evidence could be attributed to other neutral information, such as being an accountant (Kunda and Oleson, 1995). Evidence that participants were less likely to subtype if they were distracted by another task, and thus unable to judge a disconfirming target as atypical, suggests that subtyping requires considerable cognitive resources and motivation (Yzerbyt et al., 1999). More recent work has also revealed subtyping’s mechanism and its moderators. Subtyping can be driven by a motivation to embrace the stereotypes endorsed by ingroup members and to comply with the ingroup’s normative context (Carnaghi and Yzerbyt, 2006) and thus reduce the likelihood of group level social changes. Subtyping can also be moderated by preexisting intergroup attitudes. For example, facing counter-stereotypical members of the outgroup, high-prejudiced individuals subtyped positive racial outgroup members, while low-prejudiced individuals subtyped negative racial outgroup members (Riek et al., 2013).

### Good Muslim, Bad Muslim

Before situating the current research within the subtyping framework, it is helpful to outline how history has produced different stereotypes in Europe and the United States to organize perceptions of Muslims. During the colonial period, when the intercultural attitudes were developed through European travelers producing literature and art, Muslims were understood as exotic, sensuous, and depraved (Said, 1979; Ahmed, 1992). Then, throughout the twentieth century, as majority-Muslim societies attempted to form sovereign nation states, and with the onset of larger and more distant migration patterns, Muslims were now living either in newly independent countries or on the front lawns of European and U.S. cities. Much of this new period understood Muslims either as allies in a global anti-communist struggle or as resentful antagonists (Mamdani, 2002).

The sociopolitical landscape was reconfigured again after the 9/11 attacks and subsequent global “War on Terror,” offering a potent spark to solidify a stereotype of Muslims as violence-prone, anti-American extremists. In a poll conducted 6 months after 9/11, 25% of Americans believed Islam was more likely to encourage violence than other religions (Pew Research Center, 2003). By July 2003, the rate increased to 44%, indicating that the stereotype relies not only on purported evidence of violent expression but also on the contingent sociopolitical factors, with the invasion of Iraq beginning shortly before. This attribution of violence also carried gendered discourse, with militaristic and political interventions in Muslim-majority countries being justified through constructions of Islam as essentially patriarchal and homogenizing representations of Muslim women as oppressed figures in need of white, western saviors (Abu-Lughod, 2013; Wong, 2019).

Importantly, however, increasing stereotyping of Islam as encouraging violence did not coincide with a commensurate increase in explicit anti-Muslim prejudice in the United States (though Muslims are judged least favorably of any religious group). In March 2001—6 months before 9/11—45% of Americans viewed Muslim-Americans favorably (Pew Research Center, 2003). By November 2011, this favorability had *increased* to 59%, before dropping to 51% in March 2003. More recently, some argue that a general *affection* is replacing the general hostility toward Islam and Muslims, a concerted extension of liberal inclusion (with its own political costs; Shryock, 2010). So how did national sentiment toward Muslims balance this seeming contradiction, of increasing negative stereotypes without increasing explicit prejudice?

One line of research speaking to this question attempts to decouple the frequently conflated prejudice against Muslims and a secular critique of the religion itself, without reference to the adherents (Imhoff and Recker, 2012). That is, rather than a catchall “Islamophobia” that describes all hostilities for Islam and Muslims, this perspective argues for an empirical and ethical distinction between bias against the people and dispassionate disagreement with the doctrines and practices, referred to as Secular Critique of Islam. With the use of two new separate measures, two studies among non-Muslims in Germany found that Islamoprejudice was related to a separate measure of prejudice (and social dominance orientation), whereas Secular Critique was not. Islamoprejudice and Secular Critique correlated positively (*r* = 0.21) in a community sample and were unrelated in a student sample (Imhoff and Recker, 2012).

In empirically distinguishing Islamoprejudice from Secular Critique, such research ironically demonstrates, for our argument, their inextricable sociopolitical link; within everyday discourse, each way of perceiving Muslims is relevant when contrasted with the other. One study found that right-wing authoritarianism and social dominance orientation predicted the prejudicial dimension of Islamophobia but not the secular critique dimension; however, both dimensions predicted perceptions of terrorism by Muslims as a threat to the country (Italy; Tartaglia et al., 2019). In France, social scientists typically eschew the term Islamophobia for precisely this reason, because it is seen as ill-defined, too often extending to describe disparate phenomenon ranging from racism to anti-terrorism (Shryock, 2010). Perhaps not coincidentally, an aversion to the label “Islamophobia” coincides with efforts to directly restrict and control religious expression, beyond a general state secularism, a phenomenon referred to as *new secularism* (Troian et al., 2018). In studies conducted in France in the aftermath of terrorist attacks, *new secularism* (example item, “*Some* religions go against secularism,” emphasis added) can partially explain the relationship between social dominance orientation and prejudice against North Africans (Troian et al., 2018). These studies demonstrate how these dimensions of perceiving Muslims—which correspond to subtyping—are empirically distinct but politically entwined because these ways of talking and thinking about Muslims occur in tandem.

We argue that the subtyping of Muslims opens an avenue for a negative stereotype to apply to a narrow subgroup (violent extremists) while attempting to maintain a positive view of Muslims. Importantly, this social balancing act coincided with the aggressive military campaigns over the next 2 years within Muslim-majority countries (Afghanistan and Iraq) supposedly in retaliation for the 9/11 attacks. We understand this link as occurring amidst the attempt to integrate a growing Muslim population in Europe and North America while justifying the policies that target a small minority of their co-religionists, or at least coping with the realistic and symbolic threats posed by Muslims. In remarks 9 days after 9/11, then-president George W. Bush acknowledged concerns that one billion Muslims, including the many thousands living in the United States, would be subject to negative stereotypes as a result of the attacks: “The terrorists practice a fringe form of Islamic extremism that has been rejected by Muslim scholars and the vast majority of Muslim clerics; a fringe movement that perverts the peaceful teachings of Islam” (President Bush Addresses the Nation, 2001). Then, directly addressing Muslim listeners, “The terrorists are traitors to their own faith, trying, in effect, to hijack Islam itself. The enemy of America is not our many Muslim friends. It is not our many Arab friends. Our enemy is a radical network of terrorists and every government that supports them.” In the lead up to decisions of major international consequence, the leader of the most powerful military in the world simultaneously sounded alarm about a violent fringe movement and discouraged prejudicial sentiment against the religion’s peaceful adherents.

The subtyping of Muslims thus diverges from the traditional approaches to subtyping described in the previous section. Most importantly, whereas previous subtyping literature explored how biased individuals are motivated to maintain negative stereotypes of groups, the subtyping of Muslims protects a representation of Islam and Muslims as inherently *good*; the violent extremists are the exceptions to the general category. Drawing from the moral credential perspective and justification literature, we argue that this discursive framework justifies suspicion toward not only the extreme subtype, but the entire group, since the majority is acknowledged to be peaceful, absolving the group of prejudicial intent (e.g., Monin and Miller, 2001; Crandall and Eshleman, 2003). Furthermore, it places responsibility upon Muslims to disprove the public’s default suspicion, perhaps even encouraging Muslims to join in the collective cause against the perceived threat of extremism. Hawkish anti-terror policy, and the Orientalist stereotypes that accompany it, are protected from accusations of Islamoprejudice if the “good” Muslim majority is enlisted to the cause of rejecting and surveilling the “bad” Muslim minority (Mamdani, 2005). Muslim subtyping is thus a functional, context-dependent stereotype that emerges within the post-9/11 political landscape and is deployed to justify “War on Terror” policies.

### Current Research

The present studies explore how a novel form of stereotyping against Muslims is related to geopolitical attitudes, given the emergence of this subtyping within the context of the U.S.-led “War on Terror.” First, we expect to find overall support for Muslim subtyping since it is meant to protect an image of the group as inherently good. Second, we explore how Muslim subtyping is related to support for aggressive military and social policies. We predict that great endorsement of Muslim subtyping or exposure to an experimental manipulation of Muslim subtyping would predict greater levels of harsh policy. Third, the literature suggests that subtyping effects could be moderated by preexisting intergroup attitudes. We hypothesize that political orientation may moderate the relationship between Muslim subtyping and support for militaristic foreign policy. Conservatives tend to shorten cognitive thinking and are more ready to eliminate ambiguity (Petty and Jarvis, 1996; Jost et al., 2007) and thus may experience a greater threat when facing non-stereotypical evidence of Muslims than liberals. Such threat may lead conservatives to interpret such evidence as the exception that proves the “radical” Muslim subtype, rather than considering the evidence as truly counter-stereotypical. Thus, we predict that conservatives would show greater support for militaristic foreign policy in response. In addition, political conservatism is also a strong predictor of militaristic foreign policy in the literature (e.g., McCleary and Williams, 2009), and thus, responding by endorsing militaristic foreign policy is more accessible for conservatives. We tested the above hypotheses in both a correlational study (Study 1) and an experiment (Study 2).

## Study 1

The first study explored the function of subtyping by assessing its convergent and discriminant endorsement with more self-evident measures: preferences for different anti-Muslim policies and prejudice. Furthermore, this first study tested for an explicit partisan character of subtyping by measuring the relationship between subtyping and political orientation and by examining the role of political orientation in moderating the relationship between subtyping and support for anti-Muslim policies. To test for the effect of subtyping on support for policies above and beyond individuals’ levels of explicit bias, we also measured prejudice, which should be a strong positive predictor of support for interventionist policies that target Muslims. This study occurred in early 2017, when coverage of ISIS atrocities filled news coverage, and amidst heightening anti-Muslim rhetoric.

### Materials and Methods

#### Participants

We recruited 151 participants from Amazon’s Mechanical Turk. Their completion of the 10 min survey was compensated with $1 USD. Three participants failed an attention check embedded in the survey, resulting in a total sample of 148 participants (*M* = 36.72, *SD* = 12.29, range: 18–74), of whom 37.1% identified as women (one participant did not report a gender) and of whom 80.8% identified as White/Caucasian, 7.9% as Black/African American, 5.3% as Asian, 2.6% as Hispanic, and 2.0% as Native American or Pacific Islander, and 1.3% not reported.

#### Measures

Unless otherwise indicated, all self-report measures were completed with 1 (*strongly disagree*) to 7 (*strongly agree)* scales.

##### Muslim subtyping

We first constructed a five-item measure (adapted from the Secular Critique of Islam scale; Imhoff and Recker, 2012) to operationalize the preference to distinguish between two groups of Muslims as, consistent with popular portrayals, either moderate/peaceful or radical/fundamentalist/violent (α = 0.73): “Distinguishing between moderate and radical Muslims is vital to American security,” “It is wrong to ignore the threat of fundamentalist Islam,” “We should support those moderate Muslims who distance themselves from fundamentalist interpretations of Islam,” “One can fight against the political ideology of Islamic fundamentalism without having anything against non-fundamentalist Muslims,” and “I believe that most Muslims are peaceful, but to ignore the threat of radical Islamic jihad is a mistake.” We picked those items among many others in the scale that best captured a rational, open-minded distinction between Muslims who are moderate/non-fundamentalist and radical/fundamentalist.

##### Surveillance

Four items (α = 0.95) captured support for a set of tactics that would target Muslims with extra vigilance to promote American security: “I think American intelligence services should place extra effort on the surveillance of Muslim immigrants to the U.S.,” “It only makes sense to take the fact that someone is a Muslim into consideration when considering whether or not to search them at the airport,” “Even if it only helped save just one American life, spying on Muslim immigrants in the U.S. would be justified,” and “If it makes Americans safer, I think it’s justified that Muslims should come under greater scrutiny at the airport than other individuals.”

##### Anti-immigration

The average of two items captured support for suspending immigration from terror-prone, Muslim-majority regions (*r* = 0.85). With the first item, participants rated the extent to which they agreed with the statement, “I think we should suspend immigration from terror prone regions, even if it means turning away refugees from those regions.” With the second item, participants picked a point on a seven-point bipolar scale, with one anchor reading “The United States should continue to take in immigrants and refugees” and the other end “Banning people from Muslim-majority countries is necessary to prevent terrorism.”

##### Hawkish Anti-terror

Four items (α = 0.87) captured support for aggressive, militaristic policies to confront terrorism perpetrated by ISIS: “To put an end to terrorist acts by ISIS, I think it is ok to use torture,” “To put an end to terrorist acts by ISIS, I think it is OK to bomb a country if it is known to harbor ISIS terrorists,” “To put an end to terrorist acts by ISIS, I think it is OK to target supporters of ISIS with extra profiling and surveillance,” and “I support continued military efforts abroad to root out potential ISIS terrorists.”

##### Prejudice

A seven-item social distancing measure (α = 0.96) captured preference to affiliate and interact with Muslims (e.g., “Muslims are likeable people,” “I would like a Muslim to work in the same place as I do,” and “Muslims are the kind of people I tend to avoid”).

##### Political orientation

We averaged two items (α = 0.86) to capture how participants rate themselves on economic and social issues (1 = *very liberal*, 4 = *middle of the road*, 7 = *very conservative*).

##### Demographics

Participants reported their race/ethnicity, gender, education, age, and residential status.

### Results

Means, standard deviations, and correlations among variables are presented in Table 1. Consistent with the first hypothesis, participants overall endorsed Muslim subtyping (*M* = 5.35, *SD* = 1.10) to a greater extent than the neutral point of the scale (3.5), *t*(150) = 20.64, *p* < 0.001, *d* = 1.68. Generally supporting the second hypothesis, Muslim subtyping was significantly correlated with all militaristic foreign policy outcomes, except for anti-immigration.

**TABLE 1:** Descriptive results and correlations among variables.

			Correlations					
	*M*	*SD*	1	2	3	4	5	6
(1) Muslim subtyping	5.35	1.10	–					
(2) Surveillance	3.87	1.85	0.22*	–				
(3) Anti-immigration	3.64	2.12	0.09	0.81**	–			
(4) Hawkish anti-terror	4.28	1.64	0.29**	0.72**	0.64**	–		
(5) Prejudice	3.83	1.56	0.18*	0.63**	0.59**	0.36**	–	
(6) Political orientation	3.79	1.87	0.07	0.55**	0.61**	0.42**	0.48**	–

*p < 0.05, **p < 0.01.

We next conducted a series of hierarchical multiple regression analyses with subtyping and political orientation predicting support for the different policies. These models tested for the effect of subtyping as a functional stereotype in predicting anti-Muslim policies beyond the effect of explicit bias by controlling for prejudice. It was necessary to control prejudice in analyses because, as an individual difference, prejudice may be correlated with both our predictors and outcomes, serving as a confounding variable. The first set of models included each of those three predictors, and the second set tested for an additive predictive effect of the interaction between subtyping and political orientation. In all models, prejudice toward Muslims, subtyping, and conservative political orientation were significant predictors of support for surveillance, anti-immigration, and hawkish anti-terror policies.

**FIGURE 1:**
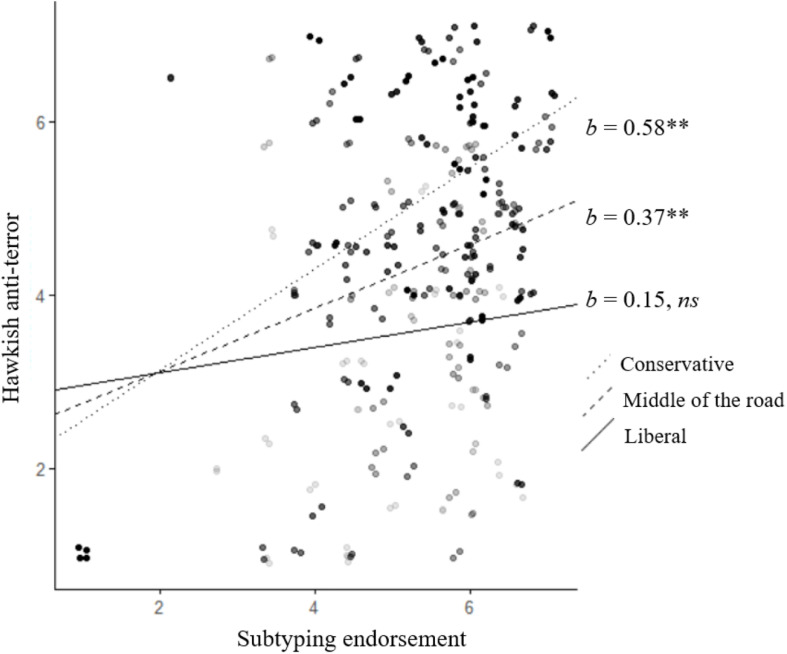
The interaction between subtyping and political orientation predicting support for hawkish anti-terror measures. Dots represent data points. Darker dots represent values of more conservative participants. **p < 0.01.

In Step 2 of all three models, the effects of subtyping and political orientation were qualified by significant *Subtyping* × *Political orientation* interactions (Table 2). We used Preacher et al.’s (2006) online tool to probe the interactions and calculate simple slopes for the relationship between subtyping and policy at different political orientations. Probing of the interaction showed that as political orientation shifted more conservatively, the relationship between subtyping and support for the policies was stronger. In the case of hawkish anti-terror, simple slope analyses showed that for liberals (i.e., at a political orientation value of 2), there was no relationship between subtyping and hawkish anti-terror, *b* = 0.15, *t* = 0.95, *p* = 0.34, 95% CI [0.01, 0.30]. However, for middle of the road and conservative participants (at political orientation scores of 4 and 6, respectively), subtyping endorsement was positively related to support for hawkish anti-terror: middle of the road participants, *b* = 0.37, *t* = 3.46, *p* = 0.009, 95% CI [0.26, 0.48], and conservative participants, *b* = 0.58, *t* = 4.31, *p* < 0.001. 95% CI [0.44, 0.72] (see Figure 1). Simple slope analyses for all three measures are summarized in Table 3.

**TABLE 2:** Prejudice, Subtyping, Political orientation, and Subtyping × Political orientation interaction predicting support for three policies.

	Surveillance		Anti-immigration		Hawkish anti-terror	
Predictor	Model 1	Model 2	Model 1	Model 2	Model 1	Model 2
Prejudice	0.67**	0.71**	0.58**	0.62**	0.31**	0.34**
Subtyping	0.50**	−0.24**	0.28*	−0.63**	0.49**	–0.08
Political	0.26**	−0.66**	0.45**	−0.69*	0.23**	–0.47
Subtyping × Political	−	0.18**	−	0.22**	−	0.13**
*R*^2^	0.57	0.62*	0.51	0.56**	0.31	0.35**

*p < 0.05, **p < 0.01.

**TABLE 3:** Results of simple slope tests for the interactions between subtyping manipulation and political orientation on support for three anti-Muslim policies.

Levels of political orientation	Surveillance	Anti-immigration	Hawkish anti-terror
Liberals	0.02	−0.32	0.15
Middle of the road	0.27*	0.04	0.37**
Conservatives	0.52**	0.40*	0.58**

Numbers represent regression coefficients. *p < 0.05, **p < 0.01.

## Study 2

Having found evidence for an association with anti-Muslim policies, the goal of Study 2 was to test for causal effects by manipulating the salience of Muslim subtyping. Given that Study 1 revealed a generally strong endorsement of Muslim subtyping (*M* = 5.35, scale from 1 to 7), we used the measure itself as a prime. Like Studies 1 and 2 examined subtyping as a predictor of relevant anti-Muslim policies.

### Materials and Methods

#### Participants

We recruited 113 undergraduate students enrolled in a psychology course to participate in the experiment in exchange for course credit. Three participants who did not complete all the measures were excluded. The final sample consisted of 110 participants (*M* = 18.95, *SD* = 0.96, range: 18–24), of whom 52.7% identified as women, and of whom 78.0% identified as White/Caucasian, 5.5% as Black/African American, 4.6% as Hispanic or Latino, 3.7% as Asian, 2.7% as multiracial, 2.7% as Native American, and 2.7% not reported.

#### Procedure

In the introduction to the study, participants in both conditions learned that they would be reading a news article, followed by questions they would answer regarding the article. This step served to set up a context for participants before they answered the Muslim-relevant questions. All participants read a news brief adapted from a CNN article titled “ISIS Fast Facts,” which described the group’s purported origins, aims, and strategies. Data collection occurred during the spring of 2017, when ISIS’s territorial control was still near its peak and news coverage regularly portrayed the group as a threat to the United States and Europe. We assumed that most participants would be familiar with ISIS, given that a previous representative survey found that 96% of U.S. respondents rated ISIS as either a “critical” or “important threat”; only 1% of respondents did not have an opinion (Gallup, 2015). After reading the article, participants completed a three-item quiz to assess reading comprehension.

Next, an order manipulation varied the salience of Muslim subtyping (see Adams, 2005, Study 3, for another example). Half of participants (*N* = 56) were randomly assigned to complete the subtyping measure from Study 1 immediately following the article and quiz. The other half of participants (*N* = 56) proceeded directly to the dependent measures, which were identical to those in Study 1 (support for surveillance, anti-immigration, and hawkish anti-terror). The subtyping measure was then completed by those participants who did not complete it earlier directly following the ISIS article.

Finally, participants reported their political orientation and demographics in identical fashion to Study 1.

### Results

Replicating results of Study 1, subtyping was endorsed in both the subtyping salient condition (*M* = 5.30, *SD* = 0.95) and the control condition (*M* = 5.38, *SD* = 0.90), and overall to a greater extent than the neutral point of the scale (3.5), *t*(111) = 21.10, *p* < 0.001, *d* = 1.99. We conducted three independent samples *t*-tests to examine the effect of the *Subtyping salient* manipulation on support for the policies. While all three tests trended in the hypothesized direction, only one effect reached statistical significance. There was no effect of the manipulation on support for anti-immigration: participants in the *Subtyping salient condition* (*M* = 3.57, *SD* = 1.82) expressed similar levels of support relative to participants in the *Control condition* (*M* = 3.09, *SD* = 1.67), *t*(109.23) = 1.46, *p* = 0.14.

There was also no effect of the manipulation on support for hawkish anti-terror: participants in the *Subtyping salient condition* (*M* = 4.89, *SD* = 1.17) expressed similar levels of support relative to participants in the *Control* condition (*M* = 4.60, *SD* = 1.23), *t*(109.74) = 1.28, *p* = 0.20.

However, there was a significant effect of the manipulation on support for surveillance: participants in the *Subtyping salient condition* (*M* = 4.71, *SD* = 1.63) expressed greater support than participants in the *Control condition* (*M* = 3.79, *SD* = 1.81), *t*(109.99) = 3.10, *p* = 0.002, *d* = 0.59.

While the study design made subtyping salient in only one condition (i.e., for only half of participants), the remaining half of participants completed the measure at the end of the study, allowing for the testing of actual endorsement of subtyping as a necessary moderator of the manipulation. To test for such an effect, we ran three models testing the effects of the manipulation, subtyping endorsement, and their interaction to predict support for the three policies.

As shown in Table 4, with the inclusion of the interaction terms, the main effect of Condition was significant in predicting greater support for each of the policies. The models predicting support for surveillance and anti-immigration did not reveal significant interactions. However, the model predicting support for hawkish anti-terror did reveal a significant interaction, a probe of which indicated that, on average, participants in the *Subtyping salient condition* expressed greater support for hawkish anti-terror measures than participants in the *Control condition*.

**TABLE 4:** Condition, Subtyping endorsement, and Condition × Subtyping interaction predicting support for three policies.

Predictor	Surveillance	Anti-immigration	Hawkish anti-terror
Condition (0 = *Control*, 1 = *Subtyping salient)*	3.57*	3.95*	3.93**
Subtyping endorsement	−0.01	0.22	0.51**
Condition × Subtyping	−0.52	−0.65	−0.68**
R^2^	0.11	0.03	0.07

*p < 0.05, **p < 0.01.

**FIGURE 2:**
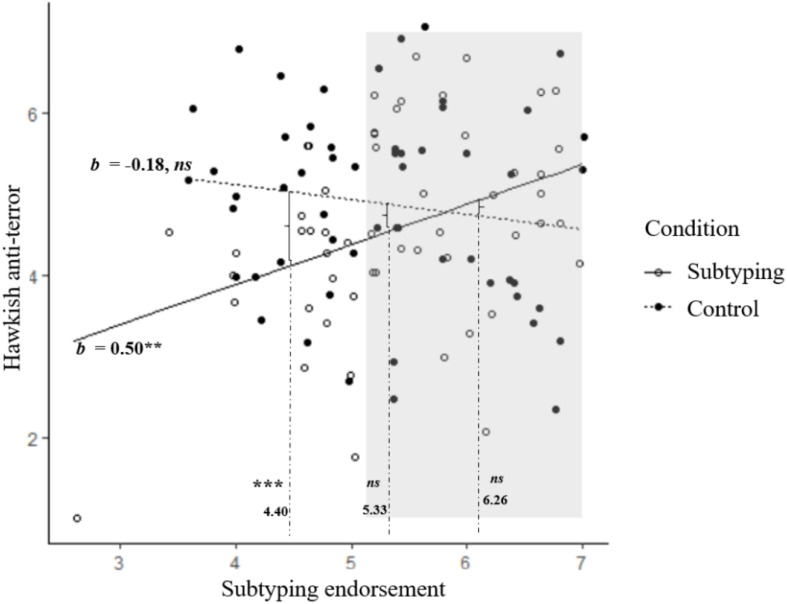
The interaction between subtyping endorsement and subtyping manipulation predicting support for the hawkish anti-terror measure. Dots represent data points. The effects of manipulated condition at different levels of subtyping endorsement are marked by brackets. With the use of the Johnson–Neyman technique, the shaded area represents the levels of subtyping at which the effect of manipulated condition was not significant in increasing support for Hawkish anti-terror policy. ***p < 0.001, **p < 0.01.

We interpreted the interaction by first treating subtyping endorsement as the moderator and the subtyping manipulation as the predictor. Simple slope analyses showed that the effect of the manipulation was significant only when subtyping endorsement was low (4.40), *b* = 0.96, *t* = 3.05, *p* = 0.003, 95% CI [0.34, 1.58], but not when subtyping endorsement was high (6.26), *b* = −0.41, *t* = −1.18, *p* = 0.239, 95% CI [−1.07, 0.27], or at intermediate level (5.33), *b* = 0.35, *t* = 1.56, *p* = 0.120, 95% CI [−0.09, 0.78] (Figure 2). These results suggest that the subtyping manipulation exerted an influence on support for hawkish anti-terror policy only when individuals previously held lower levels of such subtyping belief; for high and intermediate participants, subtyping endorsement wiped out the manipulated effect.

We also interpreted the interaction by switching the role of the variables, treating the subtyping manipulation as the moderator, and subtyping endorsement as the predictor. This simple slope analyses showed that whereas subtyping endorsement was not a significant predictor of support for the policy among participants in the *Subtyping salient condition*, *b* = −0.18, *t* = −1.09, *p* = 0.28, 95% CI [−0.51, 0.15], it was a significant predictor among participants in the *Control condition*, *b* = 0.50, *t* = 2.88, *p* = 0.005, 95% CI [0.15, 0.84] (Figure 2). This replicates what we found in Study 1 that subtyping endorsement positively predicted support for hawkish anti-terror policy.

**FIGURE 3:**
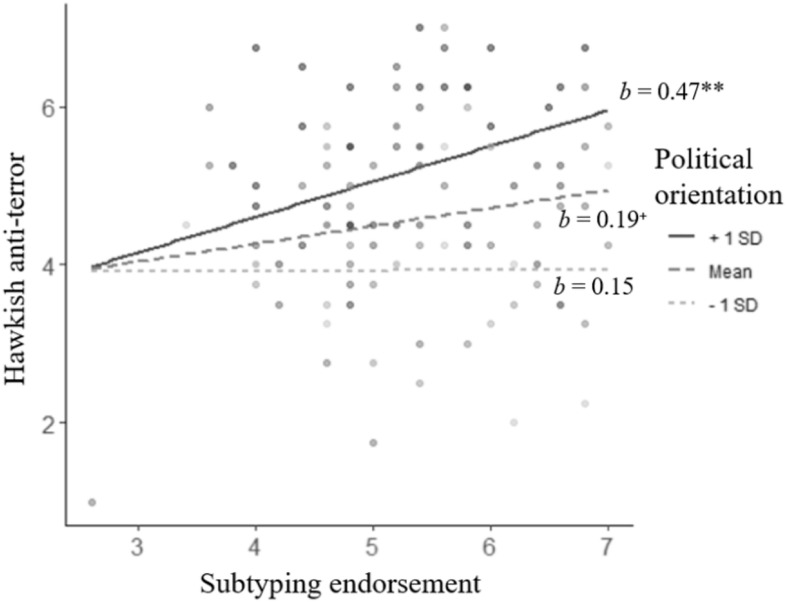
The interaction between subtyping endorsement and political orientation predicting support for hawkish anti-terror policy. Dots represent data points. ^+^*p* < 0.10, **p < 0.01.

Finally, we tested the interaction between subtyping manipulation and political orientation on support for the policies. No interactions were significant, *p*s ≥ 0.140. However, the main effects of political orientation were significant. Conservatism positively predicted support for the policies, *p*s < 0.001. However, as in Study 1, when we defined subtyping endorsement as a predictor and political orientation as a moderator, while controlling for subtyping manipulation, results generally replicated what we found in Study 1. Specifically, the interactions between subtyping endorsement and political orientation were significant on anti-immigration policy, *b* = 0.22, *t* = 2.11, *p* = 0.037, 95% CI [0.01, 0.42], on anti-terror policy, *b* = 0.14, *t* = 2.02, *p* = 0.046, 95% CI [0.002, 0.28], but not on surveillance, *b* = 0.11, *t* = 1.28, *p* = 0.203, 95% CI [−0.06, 0.27]. Simple slope analyses for the two significant measures are summarized in Table 5. In the case of anti-immigration, simple slope analyses did not reveal any significant effect of subtyping endorsement at any level of political orientation. However, for anti-terror policy, conservative participants (at political orientation score of 6), subtyping endorsement was positively related to support for hawkish anti-terror but not for liberal and middle of the road participants (see Figure 3).

**TABLE 5:** Results of subtyping endorsement’s simple slope on different levels of political orientation, Study 2.

Levels of political orientation	Anti-immigration	Hawkish anti-terror
Liberals	*b* = −0.28, *t* = −1.49, *p* = 0.139, 95% CI (−0.65, 0.09)	*b* = 0.05, *t* = 0.38, *p* = 0.703, 95% CI (−0.21, 0.30)
Middle of the road	*b* = −0.06, *t* = −0.41, *p* = 0.683, 95% CI (−0.37, 0.24)	*b* = 0.19, *t* = 1.81, *p* = 0.073, 95% CI (−0.02, 0.40)
Conservatives	*b* = 0.37, *t* = 1.47, *p* = 0.145, 95% CI (−0.13, 0.87)	*b* = 0.47, *t* = 2.74, *p* = 0.007, 95% CI (0.13, 0.82)

## General Discussion

The post-9/11 era has been fertile ground for the growth of an intercultural stereotype of Muslims as being either moderate or radical. Throughout the 2016 U.S. presidential campaign, for instance, contentious debate argued for and against using a descriptor like “radical Islam” to label Muslims who threatened the United States. Partisans of the term defended its use by arguing that it focused only on the dangerous fringe of a particular group, without encouraging any prejudice toward all Muslims. During a town hall event, then-Republican candidate Donald Trump was asked if he trusted Muslims in America. He responded: “Many of them I do. Many of them I do, and some, I guess, we don’t … We have a problem, and we can try and be very politically correct, and pretend we don’t have a problem, but … we have a major, major problem. This is, in a sense, this is a war” (Johnson and Hauslohner, 2017). The current research examined how the subtyping of Muslims into moderates and radicals, while superficially reconcilable with religious pluralism, predicts support for discriminatory policies that target Muslims.

Across one online sample and one student sample, we found that American participants overall endorse Muslim subtyping. More importantly, such endorsement translates into support for aggressive military and social policies. We witnessed this pattern when we measured Muslim subtyping in Study 1, when we manipulated Muslim subtyping in Study 2, and when we examined the subtyping endorsement’s simple slope effect within the control condition of Study 2. Subtyping endorsement was associated with greater support for surveillance policy, anti-immigration policy (when simultaneously considering the interaction effect with political orientation), and support for Hawkish anti-terror policies. Providing causal evidence, participants of Study 2 who were primed with Muslim subtyping also endorsed greater support for Hawkish anti-terror policies. Moreover, the subtyping effect on support for hawkish anti-terror policy was evident only when individuals previously held lower levels of Muslims subtyping belief. Confirming our moderation hypothesis, in both Studies 1 and 2, the relationship between subtyping endorsement and greater support for those hostile policies was particularly evident among conservatives.

Previous work on subtyping primarily focused on the process of subtyping itself (e.g., Queller and Smith, 2002; Carnaghi and Yzerbyt, 2006) and tended to focus on subtyping as an outcome. Our findings extend the literature by examining subtyping’s sociopolitical consequences. Muslim subtyping reveals how complementary stereotypes can maintain the status quo and justify ongoing harm (Jost and Kay, 2005; Kay et al., 2007). We propose that subtyping justifies support for hostile policies specifically toward “radicals,” making such behaviors more socially acceptable and even favorable, regardless of the harm caused to broader Muslim populations. Therefore, Muslim subtyping can be used and has been leveraged in U.S. political discourse as a “legitimate” tool to maneuver the vast population’s support for both domestic and foreign policies against Muslims (e.g., Muslim travel ban and Iraq war).

We also suspect that Muslim subtyping may not be unique to the United States and Europe and that the presence and effect of this discourse should be further examined in non-U.S. contexts. For example, France shares with the U.S. evocative experiences of, and responses to, domestic terrorism and a history of participation, albeit much less pronounced, in the “War on Terror” (Puar, 2007). Context-sensitive replications can uncover how the sociopolitical contingencies may produce similar results, though filtered through the laic norms that more directly racialize the Muslim minority. In China, on the other hand, in addition to promoting different norms constraining religious expression, Muslims are a longstanding domestic minority. Wei Fenghe, the Minister of National Defense of China, without even engaging in any Muslim subtyping, alleged that Xinjiang “re-education” internment camps that indoctrinate Uyghur Muslims serve to eliminate extreme values among Uyghur Muslims (Lengshanshiping, 2019).

Our findings also qualify the growing body of evidence that empirically distinguishes between prejudicial and non-prejudicial aversion to Islam and Muslims. We intentionally operationalized subtyping using items from an established measure that evidenced weak or no associations with prejudice. The present findings demonstrate that irrespective of whether it is prejudicial in nature, “culture talk” about Muslims as being either moderate or radical can unquestioningly perpetuate hostile policies (Mamdani, 2002). Future research can devise new operationalizations to test the limits of the present findings. For instance, while the current items all explicitly contrasted moderate and radical Muslims/Islam, a more conservative test of this subtyping hypothesis can include items that mention only moderate Muslims; a conceptual replication of these results with such a measure would indicate that the invoking of “moderates” alone does indeed invite thinking about “radicals” as well.

Theoretically, these findings of subtyping add to the robust literature on the adverse implications of concepts with positive guises. Such concepts include benevolent sexism (a chivalrous ideology that women should be protected by men; Glick and Fiske, 2001), the model minority myth (minorities can achieve success on their own with enough efforts; Kao, 1995), positive stereotypes (positive stereotype receivers expect being ascribed negative stereotypes; Siy and Cheryan, 2016), and patronizing forms of racism (Jackman, 1994). Frye (1983) articulates these “double-binds” as markers and mechanisms of systemic oppression. All these stereotypes send seemingly positive messages about disadvantaged groups but have subtle and insidious sociopolitical consequences—in this case, reinforcing foreign aggression and domestic discrimination.

Whereas the current research only included non-Muslim responses, future studies can investigate how Muslims perceive their group’s subtyping as well as measuring their own endorsement of subtyping. American Muslims may endorse subtyping as a way to protect positive U.S. and Muslim identities as a coping strategy to alleviate the consequences of subtyping and negative stereotypes (Branscombe et al., 1999). Muslims in non-Muslim majority settings may find those settings increasingly receptive to their expressions of moderate-ness, reinforcing the dialogic framing that anchors positive representations as existing in opposition to the negative stereotypes (Morey and Yaqin, 2011).

As the population of Muslims increases in North America and Europe, the intuition that most Muslims do not meet the violent stereotype may ironically reduce inclusion of the whole group. This occurs because the carving up of Muslims into moderates and radicals presumes that greater identification with religion is necessarily linked to violence. Ultimately, then, we would do well to release Muslims from the double-bind of subtyping and to confront political discourse that mobilizes the specter of a minority to perpetrate harm against the majority.

## Data Availability Statement

The raw data supporting the conclusions of this article will be made available by the authors, without undue reservation, to any qualified researcher.

## Ethics Statement

The studies involving human participants were reviewed and approved by the University of Kansas Institutional Review Board. The participants provided their written or electronic informed consent to participate in this study.

## Author Contributions

NH and XZ wrote the initial draft. All authors contributed to the study design, data collection, and subsequent revision of drafts.

## Conflict of Interest

The authors declare that the research was conducted in the absence of any commercial or financial relationships that could be construed as a potential conflict of interest.
